# Dexmedetomidine for prevention of postoperative pulmonary complications in patients after oral and maxillofacial surgery with fibular free flap reconstruction:a prospective, double-blind, randomized, placebo-controlled trial

**DOI:** 10.1186/s12871-020-01045-3

**Published:** 2020-05-27

**Authors:** Yun Liu, Xi Zhu, Dan Zhou, Fang Han, Xudong Yang

**Affiliations:** 1 Department of Critical Care Medicine, Peking University Third Hospital, Beijing, 100191 China; 2 Department of Anesthesiology, Peking University Hospital of Stomatology, Beijing, 100081 China

**Keywords:** Dexmedetomidine, Postoperative pulmonary complications (PPCs), Oral and maxillofacial surgery, Fibular free flap reconstruction, Tracheotomy

## Abstract

**Background:**

Postoperative pulmonary complications (PPCs) are common and significant problems for oral and maxillofacial surgery patients. Dexmedetomidine (DEX), an α_2_-adrenoreceptor agonist, has been proven having lung protection effects. However, since now, there has not been final conclusion about whether DEX can reduce the incidence of PPCs. We hypothesize that, in oral and maxillofacial surgery with fibular free flap reconstruction patients, DEX may decrease the incidence of PPCs.

**Methods:**

This was a prospective, double-blind, randomized, placebo-controlled, single-centered trial with two parallel arms. A total of 160 patients at intermediate-to-high risk of PPCs undergoing oral and maxillofacial surgery with fibular free flap reconstruction and tracheotomy were enrolled and randomized to receive continuous infusion of either DEX or placebo (normal saline). 0.4 μg/kg of DEX was given over 10mins as an initial dose followed by a maintaining dose of 0.4 μg/kg/h till the second day morning after surgery. At the same time, the normal saline was administered a similar quantity. The primary outcome was the incidence of PPCs according to Clavien-Dindo score within 7 days after surgery.

**Results:**

The two groups had similar characteristics at baseline. 18(22.5%) of 80 patients administered DEX, and 32(40.0%) of 80 patient administered placebo experienced PPCs within the first 7 days after surgery (relative risk [RR] 0.563,95% confidence interval [CI] 0.346–0.916; *P* = 0.017). In the first 7 days after surgery, the DEX group had a lower incidence of PPCs and a better postoperative survival probability (Log-rank test, *P* = 0.019), and was less prone to occur PPCs (Cox regression, *P* = 0.025, HR = 0.516). When the total dose of DEX was more than 328 μg, the patients were unlikely to have PPCs (ROC curve, AUC = 0.614, *P* = 0.009).

**Conclusions:**

For patients undergoing oral and maxillofacial surgery with fibular free flap reconstruction and tracheotomy who were at intermediate or high risk of developing PPCs, continuous infusion of DEX could decrease the occurrence of PPCs during the first 7 days after surgery and shorten the length of hospital stay after surgery, but did not increase the prevalence of bradycardia or hypotension.

**Trial registration:**

Chinese Clinical Trial Registry, www.chictr.org.cn, number: ChiCTR1800016153; Registered on May 15, 2018.

## Background

Postoperative pulmonary complications (PPCs) are a composite of the hospital-acquired respiratory events after surgery, which are one of the major causes of morbidity, mortality, and prolonged hospital stay in patients after surgery [1–3]. Oral and maxillofacial surgery is considered one of surgical factors which most likely to interfere with respiratory function and strongly linked to PPCs [4], especially radical oral and maxillofacial cancer surgery with microvascular free tissue transfer, such as fibular free flaps. Previous studies have demonstrated that 18.8 to 44.8% [4–8] of the patients undergoing head and neck surgery with free flap surgery would have PPCs, while in such kind of patients with tracheostomy the incidence of PPCs could even be 47% [6, 9]. Therefore, it is necessary to prevent and reduce the occurrence of PPCs in patients undergoing oral and maxillofacial surgery, which is a specific surgical sub-cohort within head and neck surgery, with fibular free flap reconstruction and tracheotomy.

Dexmedetomidine(DEX) is a new highly selective α_2_ adrenoceptor agonist which has anxiolysis, sedation, and modest analgesia with minimal respiratory depression effects [10] and has been widely and safely used in oral and maxillofacial surgeries [11]. Studies have proved that DEX could attenuate perioperative stress, inflammation, and protect the immune function of surgical patients [12], and can provide clinically postoperative pulmonary relevant benefits by improving oxygenation and lung mechanics [13, 14], all of which may contribute to decreased postoperative complications and improved clinical outcomes. In the last few years, a few clinical trials have evaluated the effect of DEX on PPCs [13–19]. However, the results of these studies are markedly variable and appear to be underpowered. So, since now, there has not been final conclusion about whether or not DEX can reduce the incidence of PPCs. As for the effect of DEX on PPCs in oral and maxillofacial surgeries, none of clinical trials have ever involved.

The purpose of the present study was to investigate whether DEX can reduce the incidence of PPCs during the initial 7 postoperative days in patients undergoing oral and maxillofacial surgery with fibular free flap reconstruction and tracheotomy who are at intermediate-to-high risk for PPCs.

## Methods

### Trial design

We did this prospective, double-blind, randomized, placebo-controlled, single-center, clinical trial in the department of anesthesiology of Peking University Hospital of Stomatology, a tertiary academic hospital in Beijing, China. The ethics was approved by Peking University Hospital of Stomatology Biomedical Ethics Committee (Number: PKUSSIRB-201735060) on January 26, 2018. The trial was registered with Chinese Clinical Trial Registry, www.chictr.org.cn (Number: ChiCTR1800016153) on May 15, 2018. This manuscript reporting adhered to CONSORT guidelines.

Written informed consent was obtained from all participating patients or their next of kin or legal representative who must understand the recruiter’s description of the trial. The main aim of the study was to evaluate the supremacy of the intervention. Entitled patient were enlisted and arbitrarily designed to benefit one of the interventions, DEX or placebo (normal saline).

### Randomization and blinding

A biostatistician from Peking University Third Hospital, who was independent of data management and statistical analyses, generated random numbers (in a 1:1 ratio) using the SAS 9.2 software (SAS Institute, Cary, NC, USA). The results of randomization were sealed in sequentially numbered envelopes. Throughout the survey period, enlisted patients were unpremeditated chosen to obtain DEX or placebo. A survey anesthesiologist, according to the arbitrarily series those not taking part in the survey applied the survey medicine.

The investigators, health-care team members (including the attending anesthesiologists, surgeons, nurses and the physicians for postoperative follow-up) and patients were blind to the treatment group assignment throughout the study period. In case of emergency, (such as development of severe adverse events, persistent hemodynamic instability or rapid deterioration of the patient’s clinical status), the attending anesthesiologist could request to unmask the allocation, and adjust or even stop study drug infusion if necessary. These non-blind situations were documented, but the final analyses were performed on the intention-to-treat population.

### Participants

Patients were included if they (1) were scheduled for oral and maxillofacial surgery with fibular free flap reconstruction that was expected to exceed 3 h under general anesthesia, (2) were 51 years old or over, (3) took tracheotomy before the end of the surgery, (4) had an intermediate to high risk of developing PPCs judged by Assess Respiratory Risk in Surgical Patients in Catalonia (ARISCAT) score [20](cumulative ARISCAT risk score were 26 or greater) (Additional file 1).

Patients were excluded if they met the following criteria: (1) body mass index of 35 or higher, (2) allergic to DEX, (3) recent sedatives-taking history, (4) sick sinus syndrome, or severe sinus bradycardia(< 50 beats per min[bpm]), or second degree or greater atrioventricular block without pacemaker, (5) previous lung surgery history, or severe chest wall malformation, or acute exacerbation of chronic obstructive pulmonary disease (AECOPD), or uncontrolled asthma (Asthma control test ≤18), or pulmonary artery stenosis, or pulmonary hypertension, (6) complex heart deformities, congestive heart failure, or known preoperative left ventricular ejection fraction less than 30%, (7) serious hepatic dysfunction(Child-Pugh class C), or serious renal dysfunction(requirement of renal replacement therapy), (8) a history of mental illness, (9) refused to participate in the clinical trial.

### Interventions, anesthesia and perioperative management

The study drug DEX, dexmedetomidine hydrochloride injection 2 ml: 0.2 mg (manufactured by Yangtze River Pharmaceutical (Group) Co., Ltd., Jiangsu, China), was diluted with normal saline to 50 mL (the final concentration of DEX was 4 μg/mL) by a nurse, who did not participate in the rest of the study, before administration. The study drug (diluted DEX) and placebo drug(normal saline)were all provided as clear aqueous solution in the same 50 ml injection syringes and dispensed according to the randomization results. The two drugs were given as an initial dose of 0.1 ml/kg (0.4 μg/kg of DEX in the treatment group) over 10 min followed by a maintenance dose of 0.1 ml/kg/h (0.4 μg/kg/h of DEX in the treatment group) from the beginning of anesthesia induction on the day of surgery until 0600 h on the first day after surgery.

All patients followed the similar anesthesia and perioperative management regimen. Half an hour before the beginning of the surgery, prophylactic antibiotics (mostly cefuroxime 1.5 g, the second-generation cephalosporin) were routinely administered and apply once more at the fourth hour within the operation time when the surgery time was longer than 4 hours. After surgery, routine antibiotics with cefuroxime 1.5 g twice a day and ornidazole 0.5 g twice a day for 6 days were administered. The choice and the duration of antibiotics treatment were decided according to *the Guiding Principles of Clinical Use of Antibiotics (2015 edition)* which was published by Chinese National Health and Family Planning Commission in 2015.

Perioperative monitoring included continuous 5-lead electrocardiogram, pulse oxygen saturation, noninvasive blood pressure, Train-of-Four ratio (TOF, T4/T1) for measuring the level of neuromuscular blockade, Bispectral Index (BIS) (Covidien, USA) value, end-tidal carbon dioxide concentration (EtCO_2_), airway pressure, axillary temperature, urine output. Intra-arterial pressure was also monitored through cannulation of the arteria dorsalis pedis (on the opposite of the surgical leg) immediately after anesthesia induction.

All patients were performed general anesthesia with nasotracheal intubation. Anesthesia was induced in both groups with 0.05 mg/kg midazolam, 0.3 μg/kg sufentanil, 2 mg/kg propofol, and 0.6 mg/kg rocuronium, and maintained with target-controlled infusion (TCI) of propofol (2 to 6 μg/ml plasma concentration) and remifentanil (0.5 to 6 ng/ml plasma concentration), without inhalational sevoflurane and nitrous oxide. During operation, in accordance with hemodynamic state, surgical steps and TOF ratio, additional analgesia was administered by applying boluses of sufentanil 0.1 to 0.5 μg/kg and muscle relaxation was achieved by intermittent injection of rocuronium 10 mg each time. BIS value was maintained between 40 and 60.

Volume-controlled mechanical ventilation was established with the fraction of inspiration O_2_ (FiO_2_) from 0.4 to 0.6, the tidal volume from 6 to 8 ml/kg (ideal weight), the positive end-expiratory pressure (PEEP) 5 cm H_2_O. The respiratory rate was adjusted to maintain EtCO_2_ between 35 and 45 mmHg.

Fluid management was performed according to routine practice with crystalloids - sodium lactate ringer’s injection and/or colloids - 6% hydroxyethyl starch (HES) 130/0.4 sodium injection. Packed red blood cells were transfused while the hemoglobin level was lower than 7 g/dl.

Before the end of the surgery, all patients underwent tracheotomy after spontaneous breathing recovery (TOF ratio > 0.9). After surgery, all patients were transferred to the postoperative care unit (PACU) and supervised until 0830 h on the first day after surgery before sent back to the general wards.

During the postoperative period, intravenous patient-controlled analgesia with sufentanil 1.0 ~ 1.5 μg/kg and tropisetron 10 mg was provided for up to 48 h. All patients were given aerosol inhalation with ambroxol 60 mg and hydrocortisone 4 mg three times a day before discharge and mechanical vibration sputum expectoration (TC Juhnson) three times a day for 5 days. Usually on the fifth day after surgery, the tracheostomy tube was removed after the oral and maxillofacial surgeons evaluating the situation of the airway and operation area. Other treatments including early mobilization (routinely on the fourth postoperative day), anticoagulant therapy (routinely 5 days, with aspirin or low molecular heparin), enteral and parenteral nutrition were administered according to routine practice.

The adverse events (bradycardia and hypotension) were monitored and documented throughout the period of study drug infusion. Bradycardia was defined as heart rate less than 50 beats/min or a decrease of more than 20% from baseline. Hypotension was defined as systolic blood pressure less than 90 mmHg or a decrease of more than 20% from baseline. Intervention for bradycardia included administration of medication (atropine mostly) or adjustment of study drug infusion, or both. Intervention for hypotension included intravenous fluid bolus, or administration of vasoactive drugs (ephedrine, methoxamine, etc.) or adjustment of study drug infusion. All interventions were recorded.

### Outcomes

The postoperative daily follow-up period was 7 days. Research members who were trained before the study and not involved in the clinical care of patients did the outcome assessment.

#### Primary outcome

The primary outcome was the incidence of PPCs within 7 days after surgery. PPCs was defined as any preselected complication occurred, which included respiratory infection, respiratory failure, pleural effusion, atelectasis, pneumothorax, bronchospasm, aspiration pneumonitis, pulmonary edema, pulmonary embolism, and acute respiratory distress syndrome. The diagnostic criteria of each individual PPCs were similar with those used in the previous studies [1–3](Additional file 2). We chose the Clavien-Dindo Classification [21] to categorize PPCs into five major groups (Additional file 3). In our study, PPCs of grade II or above were considered to calculate the incidence of PPCs. The diagnosis of PPCs was made by the attending medical team (anesthesiologists, Intensive Care Union physicians, or respiratory physicians). The physicians diagnosed PPCs according to patients’ medical history, clinical physical examination, conventional monitoring value, laboratory results, image examination, and so on. If a PPC occurred, the date of earliest diagnosis and the evidences according to which the diagnosis was made were documented.

#### Secondary outcomes

The secondary outcomes were as follows: (1) the time to first diagnosis of PPCs - indicated the time from end of surgery to first diagnosis of PPCs within 7 days after surgery; (2) the number of PPCs - indicated the number of diagnosed individual PPCs within 7 days after surgery; (3)the dose-effect relationship between DEX and PPCs; (4) the incidence of postoperative extrapulmonary complications - defined as complications other than PPCs that occur during operation and within 7 days after surgery, and require therapeutic intervention, included delirium – assessed by the Confusion Assessment Method for the ICU (CAM-ICU) [10], anemia - defined as hemoglobin less than 9 g/dL, extrapulmonary infection; (5) the unexpected need for secondary surgery (hematoma or vascular crisis exploration); (6) the adverse events (bradycardia, hypotension) during the period of study drug infusion; (7) length of stay in hospital after surgery; (8) 30-day all-cause mortality.

### Sample size and statistical methods

We used the excellent effect test of two groups of independent sample rate to calculate the sample size. According to the literature data [16], the sample size was calculated according to the incidence of postoperative pulmonary complications. The incidence was 3.89% in the experimental group (DEX group) and 17.99% in the control group. The class I error of hypothesis test was 0.025, the class II error was 0.2, and the proportion of sample size between the test group and the control group was 1:1. The sample size was calculated by Stata 10.0 software According to the bilateral test formula of sample size: *n* = 2 × (U _α_ + U _β_) ^2^ × P (1-P) / δ ^2^, δ was set to 0.01. The sample size of the test group was 64 and that of the control group was 64. Taking into account the dropout rate of 20%, each group requires a sample size of 76.8, so we planned to enroll 160 patients (80 for each group) in all.

We analysed outcome data and safety in the intention-to-treat population. Statistical analyses were performed on SPSS version 24.0 software (SPSS, Chicago, IL, USA) and *P* values less than 0.05 were considered to be of statistical significance. Statistical description was provided for baseline data such as demographic variables, medical history, perioperative medications, and perioperative management. For primary outcome (the incidence of PPCs with 7 days after surgery), the effect of the intervention was reported as number and percentage and estimated with relative risk and 95% confidence interval and the χ2 test for hypothesis testing. For secondary outcomes, continuous variables with normal distribution were analyzed using an unpaired t test; continuous variables with abnormal distribution or ranked data were analyzed by Mann-Whitney U test; categorical variables were analysed with the χ2 test, continuity correction χ2 test or Fisher exact test. Time-to-event results were calculated with the Kaplan-Meier estimator, and the differences between groups were assessed by the log-rank test. And, Cox regression was used for survival analysis. For dose-effect relationship, receiver operating characteristic (ROC) curve was used for calculating the P and cutoff values.

## Results

### Participant flow and recruitment

**Fig. 1:**
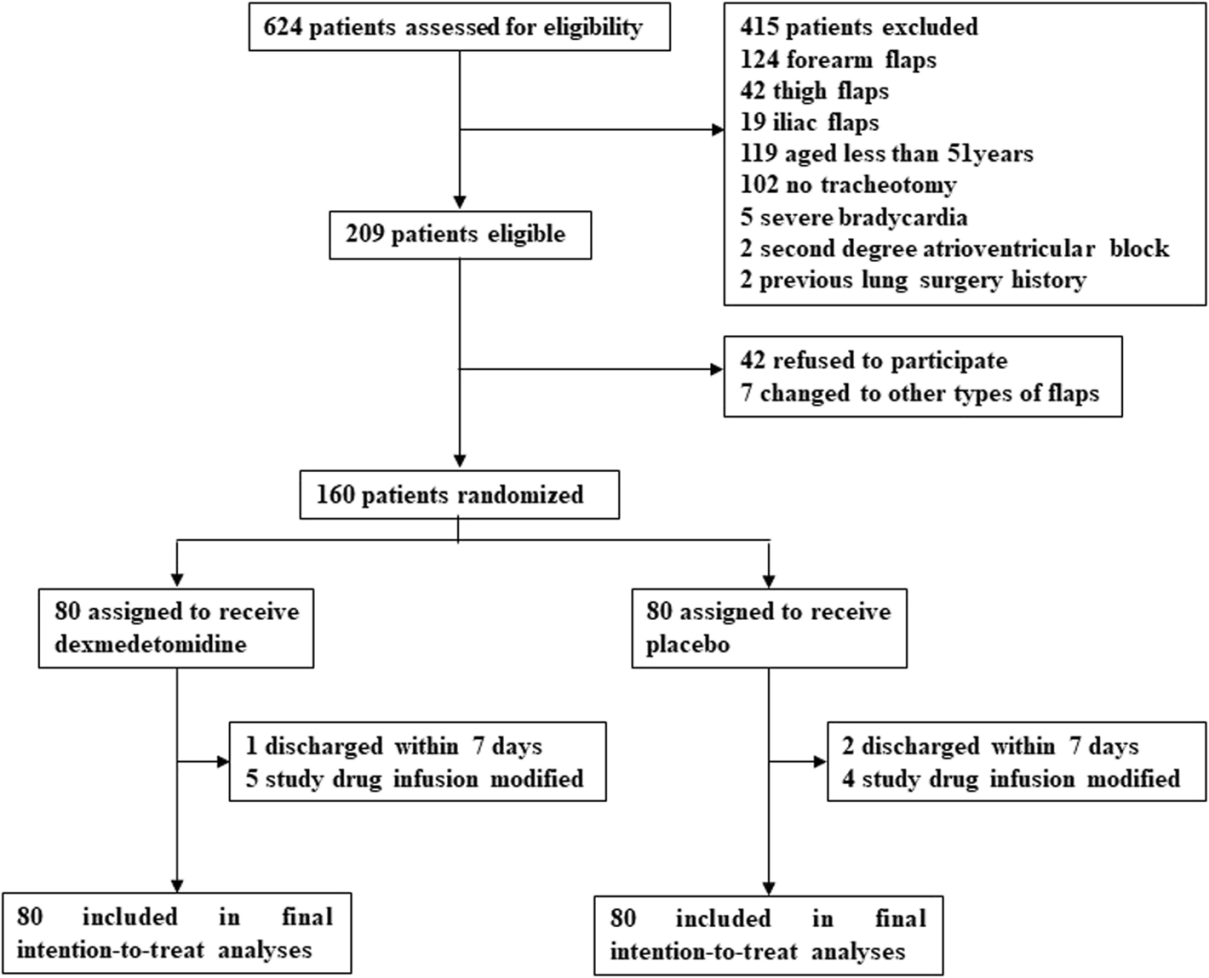
Flow Diagram of Patients Through Trial

Between September 3, 2018 and July 31, 2019, a total of 624 patients who were scheduled for oral and maxillofacial surgery with free flap reconstruction were screened for study participation; of these, 160 patients were enrolled into the study and randomly assigned to receive either DEX (*n* = 80) or placebo (n = 80). Study drug infusion was modified in 9 patients because of adverse events. Three patients were discharged from the hospital within 7 days after surgery. There were no lapses in the blinding. All patients were included in the final intention-to-treat analyses (Fig. 1). The final follow-up of the last randomized patient was finished on August 31, 2019.

### Baseline patient demographic and perioperative characteristics

Overall, the two groups were well matched for all the variables.

Both baseline patient demographic and preoperative characteristics share the same features. (Table 1).

**Table 1:** Baseline Patient Demographic and Preoperative Characteristics

Characteristic	Dexmedetomidine group (*n* = 80)	Placebo group (*n* = 80)	*P* value
Age, median (IQR), year	59 (55, 65)	62 (56, 67)	0.123
Sex, No. (%)			
Male	51 (63.8)	53 (66.3)	0.740
Female	29 (36.3)	27 (33.8)	
Hight, median (IQR), cm	168.0 (160.0, 171.5)	166.00 (160.0, 172.0)	0.832
Weight, median (IQR), kg	60.50(55.0, 69.5)	62.00 (57.5, 71.0)	0.284
BMI, median (IQR) ^a^	22.59 (20.36, 24.16)	22.90 (21.03, 25.10)	0.283
ARISCAT score ^b^			
Intermediate risk, No. (%)	77 (96.3)	79 (98.8)	0.311
High risk, No. (%)	3 (3.8)	1 (1.3)	
Mean (SD)	28.36 (4.82)	28.32 (4.81)	
ASA physical status classification, No. (%) ^c^			
1	16 (20.0)	17 (21.3)	0.216
2	61 (76.3)	63(78.7)	
3	3 (3.8)	0 (0)	
NYHA heart failure class, No. (%) ^d^			
I	47 (58.8)	51 (63.7)	0.516
II	33 (41.2)	29 (36.2)	
Tobacco use, No. (%)	31 (38.8)	29 (36.2)	0.744
Alcohol use, No. (%) ^e^	25 (31.3)	21 (26.3)	0.485
Preoperative SpO_2_, No. (%)			
≥ 96	67 (83.8)	68 (85.0)	0.975
91–95	12 (15.0)	11 (13.8)	
≤ 90	1 (1.3)	1 (1.3)	
Preoperative anemia (Hb ≤ 10 g/dl), No. (%)	9 (11.3)	5 (6.3)	0.263
Mean (SD), g/dl	13.54 (2.03)	13.72 (1.92)	
Comorbidity			
Arterial hypertension, No. (%)	27 (33.8)	24 (30.0)	0.611
Diabetes, No. (%)	9 (11.3)	12 (15.0)	0.483
Coronary artery disease, No. (%)	8 (10.0)	4 (5.0)	0.230
Arrhythmia, No. (%)	6 (7.5)	6 (7.5)	1.000
COPD, No. (%)	1 (1.3)	4 (5.0)	0.173
Asthma, No. (%)	0 (0.0)	1 (1.3)	0.316
Preoperative abnormalities on chest radiography, No. (%)	4 (5.0)	8 (10.0)	0.230
Active cancer, No. (%)	69 (86.3)	70 (87.5)	0.815

Abbreviations: *IQR* Interquartile range, *BMI* Body mass index, *ARISCAT* Assess Respiratory Risk in Surgical Patients in Catalonia, *ASA* American Society of Anesthesiology, *NYHA* New York Heart Association, *SpO*_*2*_ Oxygen saturation as measured by pulse oximetry, *Hb* Hemoglobin, *COPD* Chronic obstructive pulmonary disease

^a^ Calculated as weight in kilograms divided by height in meters squared

^b^ Score range is from 0 to 123; higher scores indicate a higher risk of postoperative pulmonary complications. Patients with scores of 26 to 44 are considered at intermediate risk; those with scores more than 44 are considered at high risk

^c^ Score range is from 1 to 6 and includes a classification for normal health as 1; mild systemic disease, 2; severe systemic disease, 3; severe systemic disease that is a constant threat to life,4. patients with scores of 5 or 6 were excluded

^d^ Score range is from I to IV; higher scores indicate a higher extent of heart failure. Patients without limitation of their ordinary physical activity are classified NYHA class I; those with slight limitation of their activity are classified as NYHA class II. Patients with scores of III or IV were excluded

^e^ Defined as more than 2 drinks per day during the past 2 weeks

For intraoperative and postoperative characteristics (Table 2, Table 3), the intraoperative dosages of propofol and remifentanil in the DEX group were significantly lower than the placebo group (*P* < 0.01), and numeric rating scale (NRS, an 11 points scale where 0 indicated the best and 10 indicated the worst) of pain for oral and maxillofacial and fibular areas on the first day after surgery were both significantly lower in the DEX group than in the placebo group (P < 0.01), as well as the sleep time on the first day after surgery in the DEX group was longer than the placebo group (P < 0.01). At the same time, intraoperative urine output and total infusion on the second day after surgery were different in the two groups, too (*P* < 0.05).

**Table 2:** Intraoperative Characteristics

Characteristic	Dexmedetomidine group	Placebo group	*P* value
	(*n* = 80)	(*n* = 80)	
Cervical lymph node dissection, No. (%)			
No	17 (21.2)	15 (18.8)	0.719
Unilateral	45 (56.3)	50 (62.5)	
Bilateral	18 (22.5)	15 (18.7)	
Duration of surgery, median (IQR), min^a^	330 (275, 382)	310 (263, 393)	0.386
Duration of anesthesia, median (IQR), min^b^	362 (302, 428)	358 (298, 442)	0.732
Duration of limb ischemia time, median (IQR), min^c^	59 (53, 71)	60 (50, 68)	0.611
Vital volume, median (IQR), ml	425 (400, 450)	425 (400, 450)	0.860
Respiratory rate, median (IQR), breaths/ min	12 (12, 13)	12 (12, 14)	0.905
Peak pressure, median (IQR), cmH_2_O			
After intubation	15 (13, 16)	14 (13, 16)	0.281
The highest during the surgery	16 (14, 18)	16 (14, 18)	0.989
Before the end of the surgery	15 (14, 17)	15 (14, 17)	0.664
FiO_2_, median (IQR)	50 (50, 50)	50 (50, 50)	0.277
Intraoperative medication, median (IQR)			
Sufentanil, μg	30 (20, 40)	30 (25, 45)	0.083
Propofol, mg	1200 (1000, 1500)	2500 (2000, 2700)	0.000
Remifentanil, μg	1450 (1000, 1980)	2625 (1700, 3000)	0.000
Dexmedetomidine, μg	188 (168, 200)	0 (0, 0)	0.000
Crystalloids, median (IQR), ml	1700 (1700, 2200)	1700 (1600, 2200)	0.947
Synthetic colloids, median (IQR), ml	500 (500, 500)	500 (500, 500)	0.149
Estimated blood loss during surgery, median (IQR), ml	300 (200, 400)	300 (200, 350)	0.508
Urine output, median (IQR)	675 (400, 900)	500 (350, 700)	0.016
Total intraoperative infusion, median (IQR), ml	1450 (1000, 1900)	1450 (1075, 1875)	0.895

Abbreviations: *FiO*_*2*_ Fraction of inspired oxygen

^a^ Calculated as the time between skin incision and closure of the incision

^b^ Calculated as the time from the start of induction to the patient leaving the operating room

^c^ Calculated as the time from the beginning of inflation to the end of exhalation of the tourniquet in the thigh

**Table 3:** Postoperative Characteristics

Characteristic	Dexmedetomidine group	Placebo group	*P* value
	(*n* = 80)	(*n* = 80)	
Length of stay in PACU, median (IQR), min	855 (675, 970)	923 (730, 1020)	0.073
Medication in PACU, median (IQR), min			
Dexmedetomidine in PACU	168 (160, 184)	0 (0, 0)	0.000
Total Dexmedetomidine on operation day ^a^	360 (338, 378)	0 (0, 0)	0.000
Sufentanil in PACU	0 (0, 0)	0 (0, 0)	0.100
Total sufentanil on operation day ^a^	30 (25, 40)	34 (25, 46)	0.070
Time with tracheotomy tube, median (IQR), d	5(5,6)	5(5,6)	0.551
Total infusion, mean (SD), ml			
The operation day ^a^	1848.65 ± 622.996	1888.44 ± 690.296	0.702
The first day after surgery	931.69 ± 1108.510	989.25 ± 1098.572	0.742
The second day after surgery	861.04 ± 954.420	868.41 ± 1162.951	0.717
NRS for oral and maxillofacial area pain, mean (SD)			
The first day after surgery	1.30 ± 1.226	3.20 ± 1.363	0.000
The second day after surgery	1.49 ± 1.534	1.53 ± 1.492	0.876
The Third day after surgery	1.39 ± 1.497	1.44 ± 1.231	0.818
NRS for fibular area pain, mean (SD)			
The first day after surgery	1.59 ± 1.357	3.71 ± 1.070	0.000
The second day after surgery	1.49 ± 1.369	1.58 ± 1.367	0.686
The third day after surgery	1.55 ± 1.457	1.43 ± 1.261	0.563
Sleep time, median (IQR), h			
The first day after surgery	6.0 (5.0, 7.0)	4.0 (3.3, 5.0)	0.000
The second day after surgery	5.0 (4.3, 6.0)	5.0 (4.0, 6.0)	0.520

Abbreviations: *PACU* Post anesthesia care unit, *NRS* Numeric rating scale

^a^ Defined the time from the beginning of the surgery to the next morning 0800 h in PACU

### Primary outcome and secondary outcomes

On the whole, PPCs within the first 7 days after surgery occurred in 18 (22.5%) of 80 patients given DEX, and in 32 (40.0%) of 80 patients given placebo (relative risk [RR] 0.563, 95% confidence interval [CI] 0.346–0.916; *P* = 0.017) (Table 4).

**Table 4:** Primary and Secondary Outcomes

Outcome	Dexmedetomidine group	Placebo group	Relative risk	*P* value
	(*n* = 80)	(*n* = 80)	(95% CI)	
Primary outcome, No. (%)				
Overall incidence of PPCs	18 (22.5)	32(40.0)	0.563 (0.346–0.916)	0.017
Respiratory infection	14 (17.5)	19 (23.8)	0.737 (0.398–1.356)	0.329
Respiratory failure	3 (3.8)	7 (8.8)	0.429 (0.115–1.599)	0.191
Pleural effusion	0 (0)	2 (2.5)	–	0.155
Atelectasis	1 (1.3)	5 (6.3)	0.200 (0.024–1.674)	0.096
Pneumothorax	0 (0)	0 (0)	–	–
Bronchospasm	0 (0)	1 (1.3)	–	0.316
Aspiration pneumonitis	0 (0)	0 (0)	–	–
Pulmonary edema	1 (1.3)	2 (2.5)	0.500(0.046–5.404)	0.560
Pulmonary embolism	1 (1.3)	1 (1.3)	1.000 (0.064–15.712)	1.000
Acute respiratory distress syndrome	1 (1.3)	0 (0)	–	0.316
Secondary outcome, No. (%)				
The time to first diagnosis of PPCs ^a^	4 (2, 5)	3 (2, 5)	–	0.928
The number of PPCs ^b^				
0.00	62 (77.5)	48 (60.0)	–	–
1.00	15 (18.8)	27 (33.8)	0.541 (0.314–0.933)	0.023
2.00	3 (3.8)	5 (6.3)	0.489 (0.123–1.954)	0.300
postoperative extrapulmonary complications ^c^				
Delirium	1 (1.3)	4 (5.0)	0.250 (0.029–2.188)	0.173
Anemia ^d^	3 (3.8)	2 (2.5)	1.500 (0.258–8.737)	0.650
Extrapulmonary infection	2 (2.5)	4 (5.0)	0.500(0.094–2.653)	0.405
Need secondary surgery ^e^	4 (5.0)	6 (7.5)	0.667 (0.196–2.273)	0.514
Adverse events				
Bradycardia ^f^	2 (2.5)	2 (2.5)	–	1
Hypotension ^g^	2 (2.5)	3 (3.8)	0.667 (0.114–3.883)	0.650
Length of stay in hospital after surgery, median (IQR), day	9 (8, 11)	10 (9, 11)	–	0.036
30-day all-cause mortality	0(0.0)	0(0.0)	–	1

Abbreviations: *PPCs* Postoperative pulmonary complications

^a^ Indicated the time from end of surgery to first diagnosis of PPCs within 7 days after surgery

^b^ Indicated the number of diagnosed individual PPCs within 7 days after surgery

^c^ Defined as complications other than PPCs that occur during operation and within 7 days after surgery, and require therapeutic intervention

^d^ Defined as hemoglobin less than 9 g/dL

^e^ Included hematoma or vascular crisis exploration within 7 days after surgery

^f^ Defined as heart rate less than 50 beats/min or a decrease of more than 20% from baseline

^g^ Defined as systolic blood pressure less than 90 mmHg or a decrease of more than 20% from baseline

Although without numerical difference, the most common PPCs was respiratory infection, accounted for 14(17.5%) patients in DEX group liken to 19(23.8%) patient in placebo group (*P* = 0.329). The incidence of the other PPCs (included respiratory failure, pleural effusion, atelectasis, pneumothorax, bronchospasm, aspiration pneumonitis, pulmonary edema, pulmonary embolism, and acute respiratory distress syndrome) was low and also without statistical difference between the two groups (*P* > 0.05).

The secondary outcomes appeared in Table 4. The incidence of one kind of PPC was less common in DEX group (RR 0.541, 95% CI 0.314–0.933; *P* = 0.023), and the length of stay in hospital after surgery was shorter in DEX group (*P* = 0.036). Nevertheless, the time to first diagnosis of PPCs, the incidence of two PPCs, the incidence of extrapulmonary complications (delirium, anemia, extrapulmonary infection), the need of secondary surgery, the incidence of adverse events (bradycardia, hypotension) and the 30-day all-cause mortality did not significantly differ between groups.

**Fig. 2:**
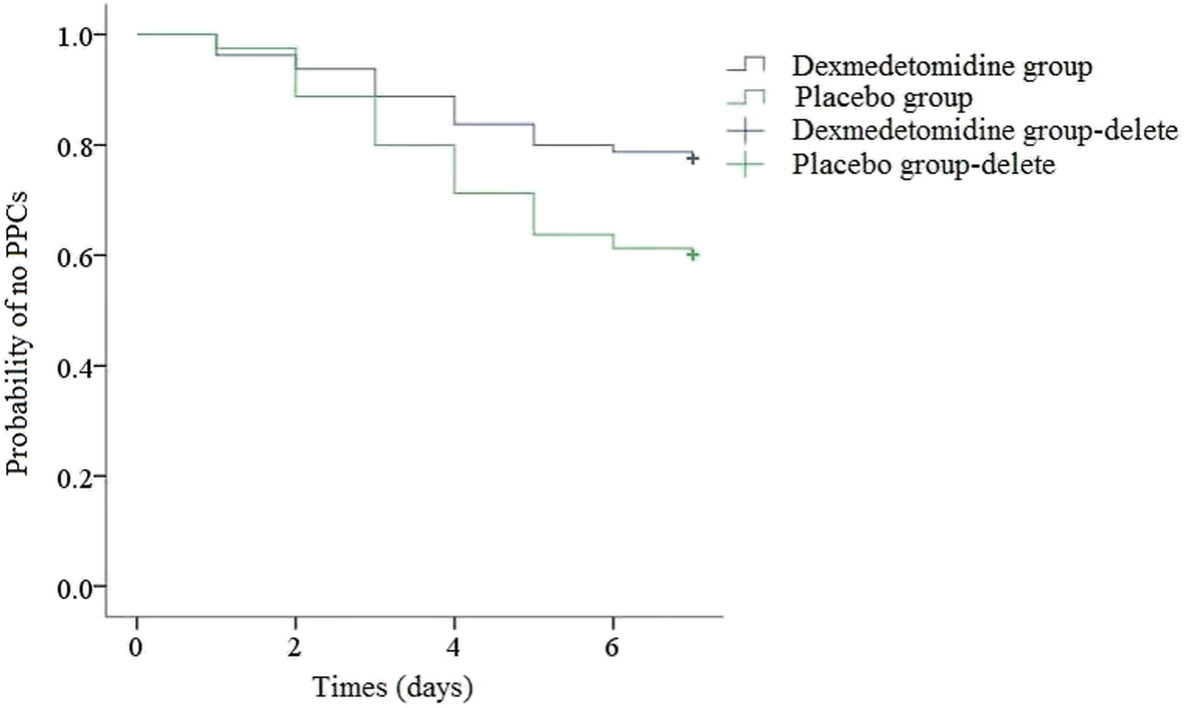
The Kaplan-Meier curve representing the time to occurrence of PPCs in the postoperative 7 days between the DEX group and the placebo group

The Kaplan-Meier curves representing PPCs in the postoperative 7 days between the DEX group and the placebo group were shown in Fig. 2. The small plus sign indicated deletion, since most of the observed objects did not have an ending at 7 days after surgery. The Log rank test results were shown in Table 5 (*P* = 0.019). Therefore, the DEX group had a lower incidence of PPCs in the first 7 days after surgery.

**Table 5:** Log rank test of the time to onset of PPCs between two groups

Groups	Mean	SE	95% Confidence Interval		P value
			Lower Bound	Upper Bound	
Dexmedetomidine group	6.212	0.191	5.837	6.588	0.019
Placebo group	5.625	0.219	5.196	6.054	
Overall	5.919	0.146	5.633	6.204	

The Cox regression results were shown in Table 6 (*P* = 0.025, HR = 0.516). Hence, within the first 7 postoperative days, the DEX group was less prone to occur PPCs.

**Table 6:** Cox regression of PPCs between two groups

Groups	B	SE	Wald	P value	HR	95.0% CI	
						Lower	Upper
Dexmedetomidine group	−0.662	0.295	5.035	0.025	0.516	0.290	0.920
Placebo group	reference						

**Fig. 3:**
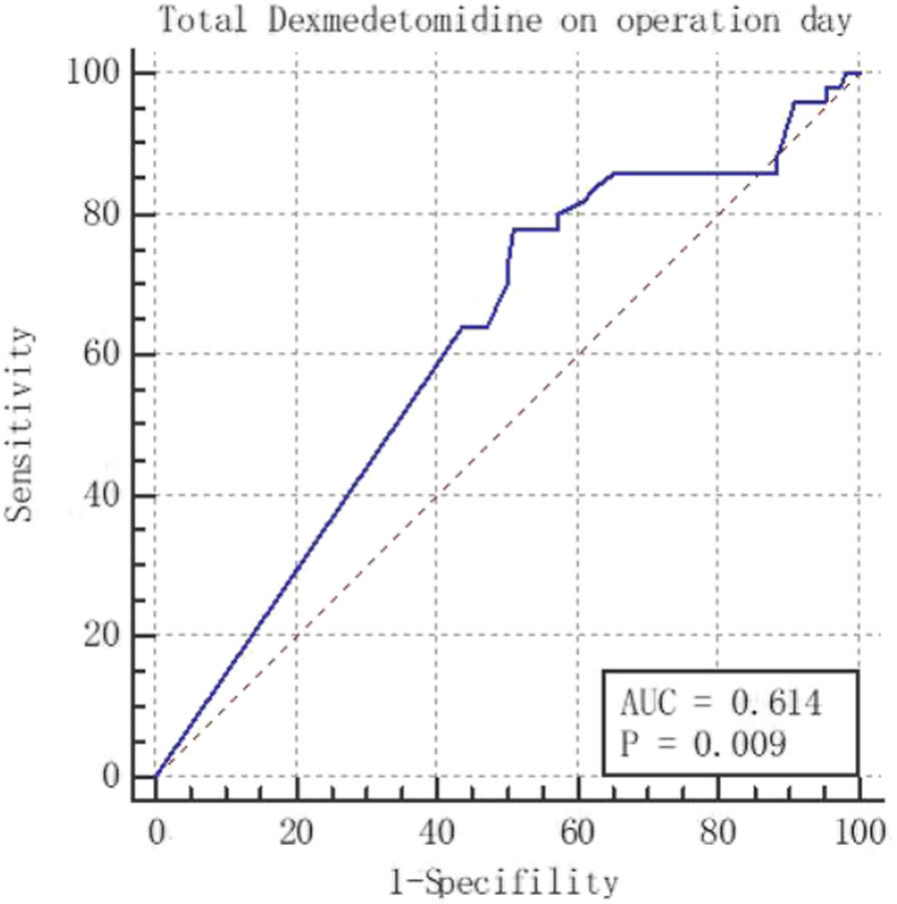
The ROC curve representing the relationship between total dose of DEX and the incidence of PPCs in the postoperative 7 days

The ROC curve results were shown in Fig. 3 and Table 7. The area under the ROC curve (AUC) was 0.614, *P* = 0.009 < 0.05, indicating that the cutoff value made by the ROC curve was statistically significant in predicting the incidence of PPCs. The sensitivity and specificity were respectively 78.00 and 49.09% and the cutoff value was 328. Hence, when the total dose of DEX on operation day was no more than 328 μg, the patients might have PPCs postoperatively, and when DEX was more than 328 μg, PPCs were unlikely to occur.

**Table 7:** The Area Under ROC curve and Youden Index

Index	Value
Area under ROC curve	0.614
Std. Error	0.0441
95% confidence interval	0.534–0.690
P value(area = 0.5)	0.0094
Youden Index	0.2709
Cutoff	≤328
Sensibility	78.00
Specificity	49.09

## Discussion

Our results suggested that DEX infusion greatly decreased the occurrence of PPCs (including respiratory infection, respiratory failure, pleural effusion, atelectasis, pneumothorax, bronchospasm, aspiration pneumonitis, pulmonary edema, pulmonary embolism, and acute respiratory distress syndrome) during the first 7 days after surgery. At the same time, DEX administration also significantly reduced the incidence of one kind of PPC and shortened the length of stay in hospital after surgery. Moreover, In the first 7 days after surgery, the DEX group had a lower incidence of PPCs and was less prone to occur PPCs. Furthermore, when the total dose of DEX was more than 328 μg, the patients were unlikely to have PPCs.

Our finding was in accordance with the previous randomized controlled clinical studies by Meiyue Liu in 2018 [16] (3.89% vs. 17.99%, *P* < 0.05) about the effects of DEX on PPCs (including hypoxemia, atelectasis and lung infection) in elderly patients undergoing spinal surgery. However, conflicting results in other studies still existed. In 2016, Su Hyun Lee [13] showed that patients with moderate COPD undergoing lung cancer surgery in DEX group had fewer incidence of PPCs, including atelectasis (0% vs. 16%, *P* = 0.110), focal lung infiltration (4% vs. 8%, *P* > 0.99)and acute lung injury (0% vs. 4%, P > 0.99) by improving oxygenation and lung mechanics, but there was no statistical difference (*P* > 0.05). In 2016, Rabie Soliman [15] found in high-risk patients undergoing aortic vascular surgery, DEX can not reduce the occurrence of PPCs (including infection and edema, *P* = 0.999), either. In 2017, Xue Li [17] found that the incidence of PPCs (including pulmonary infection, pneumothorax and pleural effusion) tended to be lower in the DEX group than in the control group (OR 0.51, 95% CI 0.26 to 1.00; *p* = 0.050) in elderly patients after cardiac surgery. In 2019, Li-Yun Zhang [18] indicated that there were no significant differences in PPCs(including atelectasis, pneumonia and air leak) between DEX and control groups (P > 0.05) in patients receiving robotic-assisted thoracic surgery.

In addition, our survey revealed the most common PPCs was respiratory infection, registering 14(17.5%) patient in DEX group in comparison with 19 (23.8%) in placebo group. Occurrence of postoperative respiratory infection was the same with the retrospective study in 2015 about 482 patients undergoing oral cancer surgery with tracheotomy [6] and the retrospective analysis of 331 cases after oral and maxillofacial surgery with or without free flap construction in 2017 [22]. Previous studies had showed that multiple variables including advanced age, male sex, poor underlying medical condition, surgery location, a higher American Society of Anesthesiologists (ASA) grade, tracheotomy and reintubation were associated with an increased risk of postoperative pneumonia [22]. All our patients were older than 51 years and underwent tracheotomy, and most of our patients were male (63.8% vs. 36.3, 66.3% vs. 33.8%, respectively), which could have been responsible for the high incidence of postoperative pneumonia in our study. But, the incidence of the other PPCs (included respiratory failure, pleural effusion, atelectasis, pneumothorax, bronchospasm, aspiration pneumonitis, pulmonary edema, pulmonary embolism, and acute respiratory distress syndrome) in our study was low. This was different from the latest two studies in JAMA 2019, which showed that respiratory failure was the most common PPC [2, 3].

Oral and maxillofacial surgery was a specific surgical sub-cohort within head and neck surgery, which was considered high risk of PPCs [23]. The fibular free flap was one of the most frequently used free flaps in oral and maxillofacial surgery, which was used for reconstruction of bony or composite defects [5]. In oral and maxillofacial surgery, patients after surgery might be highly impacted with after affect owing to swallowing and coughing, due to the body specific stance, organs of swallowing and breathing. This hinders airway which might cause the incident of PPCs as an outcome [24]. Moreover, this type of surgery had long surgical time or mechanical ventilation time (mostly more than 3 h), which might cause ventilation induced lung injury (VILI) [25], and had limb ischemia-reperfusion injury due to the use of the tourniquet in the thigh area, which might induce remote lung damage [26]. Except that, after the type of surgery, due to the microvascular reconstruction technique in the neck region, patients are required to stay lying in bed restraining lots of neck movement for at least 3 days after surgery, which might bring about respiratory muscle complications and mouth ejection and even PPCs [27]. Thus, one might expect a high rate of PPCs after oral and maxillofacial surgery, which had been proven in previous studies [5, 7, 8]. In our study, PPCs developed in 40.0% (32 of 80) of oral and maxillofacial surgery with tracheotomy patients in the placebo group, largely in agreement with the prospective, randomized, controlled trial study about major head and neck surgery with tracheostomy (47%) [9], but much higher than the retrospective analysis of 648 cases about major oral and maxillofacial surgery with microvascular reconstruction (18.8%) [5]. To this conflict, we considered the risk of tracheotomy might be the main cause, but this needed more proofs. On the other hand, our data further supported the evidence that oral and maxillofacial surgery was associated with a high risk of PPCs.

Although DEX was generally well tolerated, it could cause concentration related adverse circulatory effects (e.g. bradycardia and hypotension) [28]. However, the relationship between the dose use/ the mode of DEX administration (a single-bolus injection, a continuous infusion, or both in combination) and cardiac side-effects had not been established [12]. In our study, the median (IQR) of age of the two groups was 59 (55,65) and 62 (56,67) years, so in consideration of concentration related adverse circulatory effects for elderly patients, we chose a 0.4 μg/kg infusion over 10 min, followed by a dose of 0.4 μg/kg/h to maintain the anesthesia. The similar low DEX infusion dosing could be found in many other studies [10, 29–31]. And our study also indicated that this dose of DEX continuous infusion did not increase the prevalence of bradycardia or hypotension, so we thought that our DEX dose was proper for safety evaluation.

A systematic review and meta-analysis in BMJ indicated that postoperative pulmonary complications typically took place within the first week after surgery [32], and it had been proved that the time between surgery and the first postoperative pulmonary complication was about 3 (2 to 6) days [33]. These conclusions were similar in our study, in which the first time of diagnosis of PPCs was 4 (2 to 5) days in DEX group and 3 (2 to 5) days in control group. We assumed that DEX infusion could alleviate the lung injury during the infusion time by reducing inflammation and stress, as well as protecting the immune function [34]. But the effects of DEX on clinical outcomes, such as PPCs, would gradually emerge after DEX termination. There had been a great number of clinical studies which demonstrated that perioperative DEX administration had benefits about long term (e.g. postoperative 30 days [17], even 3 years [35]) clinical outcomes. In consequence, we chose a time frame of postoperative 7 days about primary outcome assessment. And this could explain why the Kaplan-Meier analysis of the present RCT the most profound difference between groups occurred after the postoperative day 4 (long after DEX termination).

It started becoming clear that there were contrast between those above surveys looking back to the overview of contrast and similarities, including the study population, the surgery type, the diagnostic criteria of PPCs, the observation time of PPCs, the dose of DEX, and so on. Therefore, our study was the first prospective clinical trial about the relationship between DEX and PPCs in patients undergoing oral and maxillofacial surgery with microvascular reconstruction and tracheotomy.

So far, the evidence on the accurate mechanisms of the effects about DEX on PPCs remained poorly understood, because of the complex etiology and pathophysiology and different diagnosis criteria of PPCs. A number of previous animal and clinical studies had tried to reveal the various mechanisms which might contribute to the lung protective effect of DEX. First, DEX, as a new highly selective α_2_ adrenergic receptor agonist, could alleviate lung injury by improving ventilation/perfusion ratio and oxygenation by directly stimulating α_2B_ receptors in lung vascular smooth muscles [14]. Second, DEX had direct protective effects on airway. An animal experiment in dogs reported that DEX had a bronchodilator effect in histamine-mediated bronchospasm [36], as well as a study in guinea pig improved that DEX had a direct airway smooth muscle effect and an underlying mechanism for cough suppression by inhibiting acetylcholine releasing from cholinergic nerves [37]. Third, inflammation was an important cause of lung injury. Various studies had showed that DEX could suppress systemic inflammatory processes by downregulation the signaling pathway of HMGB1-TLR4-MyD88-MARK- NF-κB and inflammatory mediators of IL-1, IL-4, IL-6, IL-8 and TNF-α, etc. [34, 38]by activating α_2_ adrenergic receptors and stimulating the vagus nerve via a vagal and α_7_ nicotinic acetylcholine receptor-dependent mechanism [39]. Fourth, DEX had been demonstrated to have lung protective effects by reducing dead space ventilation, increasing dynamic compliance [13], so this point also might be one of the reasons why DEX could decrease PPCs in our study. Fifth, propofol, which was the main anesthetic during operation in anesthesia maintain period in our study, was reported to have a higher rate of PPCs compared with inhaled anesthetics sevoflurane [40]. In our study, the amount of propofol was significantly lower in the DEX group than in the placebo group, so we might conclude that, after intravenous infusion of DEX, the decreasing demand for propofol had a beneficial effect of reducing the incidence of PPCs. Six, our results showed a significant difference in intraoperative remifentanil consumption between the two groups, which could be explained that the lower opioid consumption in the DEX group was caused by the analgesic effect of DEX. Remifentanil was an ultra-short-acting, potent opioid analgesic. Previous studies showed that remifentanil could attenuate inflammation and acute lung injury through signaling pathway [41, 42]. So, in our study, the lower occurrence of PPCs in DEX group might be in connection with the lessened amount of remifentanil. Seven, Ahmed Hasanin in 2018 [43] supposed in his paper that the improvement of lung mechanics might be due to the potential better sedation state of DEX administration to have better relaxation of the chest wall, but this needed further proofs. However, overall speaking, all those above assumptions required further evaluation in more studies with higher level of evidence.

Apart from DEX-induced improvements in PPCs and postoperative length of stay in hospital, our study also found that on the first day after oral and maxillofacial surgery, DEX alleviated the subjective pain in both oral and maxillofacial area and fibular area, and increased the objective sleep time. These results were similar with the clinical trial in Lancet by Su Xian in 2016 [10]. The analgesia effect of DEX was worked by acting on the α_2_ adrenergic receptors in the spinal cord [44]. The hypnotic properties of DEX was exerted by activating the endogenous sleep-promoting pathway and producing a stage II non-rapid eye movement sleep-like state [45].

Besides, previous study had confirmed that prophylactic DEX significantly decreased the occurrence of delirium during the first 7 days after non-cardiac surgery [10]. However, in our study, we found that patients in the DEX group had less delirium incidence than patients in the placebo group (1.3% vs. 5.0%; RR 0.250, CI 0.029–2.188; *P* = 0.173), but without statistical difference. We inferred the reason might be our sample size was not big enough considering the low incidence of postoperative delirium in our study population.

The survey had a lot of limitations. (1) The study was only designed to investigate differences in postoperative clinical practice (incidence of PPCs), but lack of research of the effect of DEX on biological markers (in plasma or bronchoalveolar lavage fluid) about lungs damages and indicators about respiratory dynamics throughout the perioperative time. (2) Regarding the high hospital cost, serum concentration of DEX wasn’t calculated. (3) This trial only studied one kind of transfusion speed and one administration way of DEX, so different transfusion speeds and different administration ways should be further investigated. (4) The finding of ROC curve analysis was not that robust, so the finding of the dose-effect relationship between DEX and PPCs can be considered valid only for this specific clinical setting. (5) Since our study was the first clinical trial to evaluate the influence of DEX on the incidence of PPCs in patients undergoing oral and maxillofacial surgery, and we did not conduct a pilot study, the sample size calculation which we referred to the relevant data of patients undergoing spinal surgery might not be very accurate. Fortunately, the positive result was obtained in this study. However, future larger sample size clinical trials are needed to verify our findings. (6) Due to the complex and various definition of PPCs and the hospital where we carried out this study is a specialized Stomatological hospital, the improper estimation of PPCs did exist. Therefore, further research in the future will take the above factors into account.

## Conclusion

For patients undergoing oral and maxillofacial surgery with fibular free flap reconstruction and tracheotomy who were at intermediate or high risk of developing PPCs, continuous infusion of DEX could decrease the occurrence of PPCs during the first 7 days after surgery and shorten the length of hospital stay after surgery, but did not increase the prevalence of bradycardia or hypotension. In consideration of the limitations in our study, a larger sample size may be required to verify the difference in the future.

## Supplementary information

**Additional file 1.** The Seven ARISCAT Risk Predictors.

**Additional file 2.** Definitions of Postoperative Pulmonary Complications.

**Additional file 3.** Criteria of grade of PPCs according to the Clavien-Dindo classification.

## Data Availability

The datasets generated and/or analyzed during the current study will be available from the corresponding author on a reasonable request.

## Abbreviations

PPCs: Postoperative pulmonary complications
DEX: Dexmedetomidine
RR: Relative risk
CI: Confidence interval
ARISCAT: Assess Respiratory Risk in Surgical Patients in Catalonia
AECOPD: acute exacerbation of chronic obstructive pulmonary disease
TOF: Train-of-Four
BIS: Bispectral Index
EtCO_2_: End-tidal carbon dioxide concentration
TCI: Target-controlled infusion
FiO_2_: Fraction of inspiration O_2_
PEEP: Positive end-expiratory pressure
HES: Hydroxyethyl starch
PACU: Postoperative care unit
NRS: Numeric rating scale
VILI: Ventilation induced lung injury
ASA: American Society of Anesthesiologists
CAM-ICU: Confusion Assessment Method for the ICU
ROC: Receiver operating characteristic
AUC: Area under the ROC curve
IQR: Interquartile range
BMI: Body mass index
NYHA: New York Heart Association
SpO2: Oxygen saturation as measured by pulse oximetry
Hb: Hemoglobin
COPD: Chronic obstructive pulmonary disease
