# Supplementary material for: Dexmedetomidine for prevention of postoperative pulmonary complications in patients after oral and maxillofacial surgery with fibular free flap reconstruction:a prospective, double-blind, randomized, placebo-controlled trial
Source: BMC Anesthesiol. 2020 May 27;20:127. doi: 10.1186/s12871-020-01045-3 (PMC7251859; doi:10.1186/s12871-020-01045-3)
Supplement: Supplementary file 1 — Additional file 1. The Seven ARISCAT Risk Predictors. [file 12871_2020_1045_MOESM1_ESM.docx]

**The Seven ARISCAT Risk Predictors ^a^**

| Independent Predictors of Risk for PPCs | Score |
| --- | --- |
| Age, years |  |
| ≤ 50 | 0 |
| 51-80 | 3 |
| > 80 | 16 |
| Preoperative SpO_2_, % |  |
| ≥ 96 | 0 |
| 91-95 | 8 |
| ≤90 | 24 |
| Respiratory infection in the last month |  |
| No | 0 |
| Yes | 17 |
| Preoperative anemia(Hb≤10g/dl) |  |
| No | 0 |
| Yes | 11 |
| Surgical incision |  |
| Peripheral | 0 |
| Upper abdominal | 15 |
| Intrathoracic | 24 |
| Duration of surgery, hours |  |
| ≤2 | 0 |
| 2-3 | 16 |
| >3 | 23 |
| Emergency procedure |  |
| No | 0 |
| Yes | 8 |

Abbreviations: ARISCAT, Assess Respiratory Risk in Surgical Patients in Catalonia; PPCs, Postoperative Pulmonary Complications; SpO_2_, oxygen saturation as measured by pulse oximetry; Hb, hemoglobin.

^a^ Score range is from 0-123; higher scores indicate a higher risk of PPCs. Patients with scores less than 26 are considered at low risk; those with 26 or greater are considered at intermediate risk and those with a score greater than 44 are considered at high risk.
