# Supplementary material for: Dexmedetomidine for prevention of postoperative pulmonary complications in patients after oral and maxillofacial surgery with fibular free flap reconstruction:a prospective, double-blind, randomized, placebo-controlled trial
Source: BMC Anesthesiol. 2020 May 27;20:127. doi: 10.1186/s12871-020-01045-3 (PMC7251859; doi:10.1186/s12871-020-01045-3)
Supplement: Supplementary file 2 — Additional file 2. Definitions of Postoperative Pulmonary Complications. [file 12871_2020_1045_MOESM2_ESM.docx]

**Definitions of Postoperative Pulmonary Complications**

| Complications | Definition |
| --- | --- |
| Respiratory infection | Receiving antibiotics for a suspected respiratory infection and met at least one of the following criteria: new or changed sputum, new or changed lung opacities, fever, leukocyte count >12 × 10^9^/L |
| Respiratory failure | PaO_2_ < 60 mmHg on room air, a ratio of PaO_2_ to inspired oxygen fraction < 300, or arterial oxyhemoglobin saturation measured with pulse oximetry < 90% and requiring oxygen therapy |
| Pleural effusion | Chest X-ray demonstrating blunting of the costophrenic angle, loss of the sharp silhouette of the ipsilateral hemidiaphragm in upright position, evidence of displacement of adjacent anatomical structures, or (in supine position) a hazy opacity in one hemithorax with preserved vascular shadows |
| Atelectasis | Lung opacification with a shift of the mediastinum, hilum, or hemidiaphragm toward the affected area, and compensatory overinflation in the adjacent nonatelectatic lung |
| Pneumothorax | Air in the pleural space with no vascular bed surrounding the visceral pleura |
| Bronchospasm | Newly detected expiratory wheezing treated with bronchodilators |
| Aspiration pneumonitis | Acute lung injury after inhalation of regurgitated intragastric contents |
| Pulmonary edema | Defined as diffuse alveolar interstitial infiltrates with dyspnea and rales related to left ventricular failure, confirmed by one of the following: echocardiography, pulmonary catheter, or clinical improvement with specific treatment |
| Pulmonary embolism | Patients may suffer from sudden shortness of breath or fast breathing; sudden chest pain that is worse when you take a deep breath; fast heartbeat; fever and coughing up blood; bluish nails; cold, pale, clammy skin; fainting. Tests for diagnosing include: d-Dimer test, arterial blood gas study, chest X-rays, ultrasound of the legs, electrocardiogram (EKG), computerized tomography (CT) scan, et al. |
| ARDS | Within 1 week of a known clinical insult or new/worsening respiratory symptoms; chest imaging showed bilateral opacities—not fully explained by effusions, lobar/lung collapse, or nodules; respiratory failure not fully explained by cardiac failure or ﬂuid overload;  Need objective assessment (e.g., echocardiography) to exclude hydrostatic edema if no risk factor present. |

*Abbreviations: PaO_2_, partial pressure of oxygen in arterial blood; ARDS, acute respiratory distress syndrome.*
