# Supplementary material for: Dexmedetomidine for prevention of postoperative pulmonary complications in patients after oral and maxillofacial surgery with fibular free flap reconstruction:a prospective, double-blind, randomized, placebo-controlled trial
Source: BMC Anesthesiol. 2020 May 27;20:127. doi: 10.1186/s12871-020-01045-3 (PMC7251859; doi:10.1186/s12871-020-01045-3)
Supplement: Supplementary file 3 — Additional file 3. Criteria of grade of PPCs according to the Clavien-Dindo classification. [file 12871_2020_1045_MOESM3_ESM.docx]

**Criteria of grade of PPCs according to the Clavien-Dindo classification**

| Complications | I | II | IIIa | IIIb | IVa | IVb | V |
| --- | --- | --- | --- | --- | --- | --- | --- |
| Respiratory infections | Clinical observation or diagnostic evaluation only; intervention not indicated except for nebulizers, expectorants, or lung physiotherapy (e.g., postural drainage) | Medical management indicated (e.g., antibiotics) | Intervention not under general anesthesia (e.g., bronchoscopic aspiration, tracheal puncture) | Intervention under general anesthesia (e.g., tracheostomy under general anesthesia or sedation) | Mechanical ventilation indicated | Sepsis or multiple organ failure | Death |
| Respiratory failure | _ | _ | _ | _ | Mechanical ventilation indicated | Sepsis or multiple organ failure | Death |
| Pleural effusion | Clinical observation or diagnostic evaluation only; intervention not indicated (drainage only through existing drainage tube) | Medical management indicated (e.g., diuretics) | Intervention not under general anesthesia (e.g., Image-guided drain placement or thoracentesis including drain replacement indicated) | Intervention under general anesthesia indicated | Mechanical ventilation indicated | Multiple organ failure | Death |
| Atelectasis | Clinical observation or diagnostic evaluation only; intervention not indicated, except for nebulizers, expectorants, or lung physiotherapy (e.g., postural drainage) | Medical management indicated (e.g., antibiotics) | Intervention not under general anesthesia (e.g., bronchoscopic aspiration, tracheal puncture) | Intervention under general anesthesia (e.g., tracheostomy under general anesthesia or sedation) | Mechanical ventilation indicated | Sepsis or multiple organ failure | Death |
| Pneumothorax | Clinical observation or diagnostic evaluation only; intervention not indicated (drainage only through existing drainage tube) | _ | Intervention not under general anesthesia (e.g., closed drainage of thoracic cavity or thoracentesis including drain replacement indicated) | Intervention under general anesthesia indicated | Mechanical ventilation indicated | Multiple organ failure | Death |
| Bronchospasm | Clinical observation or diagnostic evaluation only; intervention not indicated except for nebulizers (bronchodilators not included), expectorants, or lung physiotherapy (e.g., postural drainage) | Medical management indicated (e.g., bronchodilators) | _ | _ | Mechanical ventilation indicated | Multiple organ failure | Death |
| Pulmonary edema | Clinical observation or diagnostic evaluation only; intervention not indicated | Medication  management | Intervention not under general anesthesia | Intervention under general anesthesia indicated | Mechanical ventilation indicated | Sepsis or multiple organ failure | Death |
| Pulmonary embolism | Clinical observation or diagnostic evaluation only; intervention not indicated | Medical management indicated (e.g., anticoagulants) | Invasive treatment indicated (e.g., thrombus ablation via catheter, IVC filter) | Intervention under general anesthesia indicated (pulmonary artery thrombectomy) | Single organ failure caused by thrombi (e.g., lung, brain, heart) | Multiple organ failure caused by thrombi (e.g., lung, brain, heart) | Death |
